# Predictive models of RNA degradation through dual crowdsourcing

**Published:** 2021-10-14

**Authors:** Hannah K. Wayment-Steele, Wipapat Kladwang, Andrew M. Watkins, Do Soon Kim, Bojan Tunguz, Walter Reade, Maggie Demkin, Jonathan Romano, Roger Wellington-Oguri, John J. Nicol, Jiayang Gao, Kazuki Onodera, Kazuki Fujikawa, Hanfei Mao, Gilles Vandewiele, Michele Tinti, Bram Steenwinckel, Takuya Ito, Taiga Noumi, Shujun He, Keiichiro Ishi, Youhan Lee, Fatih Öztürk, Anthony Chiu, Emin Öztürk, Karim Amer, Mohamed Fares, Eterna Participants, Rhiju Das

**Affiliations:** 1 Department of Chemistry, Stanford University, Stanford, California 94305, USA; 2 Department of Biochemistry, Stanford University, California 94305, USA; 3 NVIDIA Corporation, Santa Clara, California 95051; 4 Kaggle, San Francisco, California 94107; 5 Eterna Massive Open Laboratory; 6 Department of Computer Science and Engineering, State University of New York at Buffalo, Buffalo, New York, 14260, USA; 7 High-flyer AI, Hangzhou, Zhejiang, China, 310000; 8 NVIDIA Corporation, Minato-ku, Tokyo 107-0052, Japan; 9 DeNA, Shibuya-ku, Tokyo 150-6140, Japan; 10 Yanfu Investments, Shanghai, China, 200000; 11 IDLab, Ghent University, Technologiepark-Zwijnaarde, Gent, Belgium, B-9052; 12 College of Life Sciences, University of Dundee, Dundee DD1 4HN, United Kingdom; 13 Universal Knowledge Inc., Tokyo 150-0013, Japan; 14 Keyence Corporation, 1-3-14, Higashi-Nakajima, Higashi-Yodogawa-ku, Osaka, 533-8555, Japan; 15 Department of Chemical Engineering, Texas A&M University, College Station, TX 77843; 16 Rist Inc, Meguro-ku, Tokyo 153-0063, Japan; 17 Kakao Brain, Seongnam, Gyeonggi-do, Republic of Korea; 18 H2O, Istanbul, 3400, Turkey; 19 Clover Health, Hong Kong, 999077, PRC; 20 Afiniti, Istanbul, 3400, Turkey; 21 Center for Informatics Science, Nile University, Sheikh Zayed, Giza, Egypt, 12588; 22 National Research Centre, Dokki, Cairo, Egypt, 12622; 23 Department of Physics, Stanford University, California 94305, USA

## Abstract

Messenger RNA-based medicines hold immense potential, as evidenced by their rapid deployment as COVID-19 vaccines. However, worldwide distribution of mRNA molecules has been limited by their thermostability, which is fundamentally limited by the intrinsic instability of RNA molecules to a chemical degradation reaction called in-line hydrolysis. Predicting the degradation of an RNA molecule is a key task in designing more stable RNA-based therapeutics. Here, we describe a crowdsourced machine learning competition (“Stanford OpenVaccine”) on Kaggle, involving single-nucleotide resolution measurements on 6043 102–130-nucleotide diverse RNA constructs that were themselves solicited through crowdsourcing on the RNA design platform Eterna. The entire experiment was completed in less than 6 months. Winning models demonstrated test set errors that were better by 50% than the previous state-of-the-art DegScore model. Furthermore, these models generalized to blindly predicting orthogonal degradation data on much longer mRNA molecules (504–1588 nucleotides) with improved accuracy over DegScore and other models. Top teams integrated natural language processing architectures and data augmentation techniques with predictions from previous dynamic programming models for RNA secondary structure. These results indicate that such models are capable of representing in-line hydrolysis with excellent accuracy, supporting their use for designing stabilized messenger RNAs. The integration of two crowdsourcing platforms, one for data set creation and another for machine learning, may be fruitful for other urgent problems that demand scientific discovery on rapid timescales.

## Introduction

The chemical instability of RNA sets a fundamental limit on the stability of RNA-based therapeutics such as mRNA-based vaccines^1–4^. Better methods to develop thermostable RNA therapeutics would allow for increasing their potency, increasing the equitability of their distribution, and reducing their cost. A key prediction task underpinning the design of thermostable RNA therapeutics is the prediction of RNA hydrolysis from sequence and structure. Previous models for RNA degradation have assumed that the probability of any RNA nucleotide linkage being cleaved is proportional to the probability of the 5’ nucleotide being unpaired^5^. Computational studies with this model suggested that at least a two-fold increase in stability could be achieved through sequence design, while maintaining a wide diversity of sequences and features related to translatability, immunogenicity, and global structure^6^. However, it is unlikely that degradation depends only on the probability of a nucleotide being unpaired: local sequence-and structure-specific contexts may vary widely.

We wished to understand the maximum predictive power achievable for RNA degradation on a short timescale for model development. To do this, we combined two crowdsourcing platforms: Eterna, an RNA design platform, and Kaggle, a platform for machine learning competitions. Eterna has previously been able to solve near-intractable problems in RNA design^7, 8^, and the diversity of resulting structures on its platform have more recently contributed to advancing RNA secondary structure prediction^9^. We reasoned that crowdsourcing the problem of obtaining data on a wide diversity of sequences and structures would rapidly lead to a diverse dataset, and that crowdsourcing the second problem of obtaining a machine learning architecture would result in a model capable of expressing the resulting complexity of sequence- and structure-dependent degradation patterns. We hypothesized this “dual crowdsourcing” would lead to stringent and independent tests of the models developed, minimizing interplay between the individuals designing the constructs to test (Eterna participants) and the individuals building the models (Kaggle participants) and leading to better generalizability on independent data sets.

The resulting models were subjected to two blind prediction challenges: the first was in the context of the Kaggle competition, where the data that Kaggle participants were scored on was not produced until after the challenge was set to the participants. The resulting models also demonstrated increased predictive power in a completely independent challenge of predicting the overall degradation of full-length mRNAs encoding the protein nanoluciferase, which were experimentally tested in a different wet lab pipeline. The models therefore appear immediately useful for guiding design of low degradation mRNA molecules. Analysis of model performance suggests that the task of predicting RNA degradation patterns is limited by both the amount of data available as well as the accuracy of the structure prediction tools used to create input features. Further developments in experimental data and secondary structure prediction, when combined with network architectures such as those developed here, will further advance RNA degradation prediction and therapeutic design.

## Results

### Dual-crowdsourced competition design and assessment.

**Figure 1.**
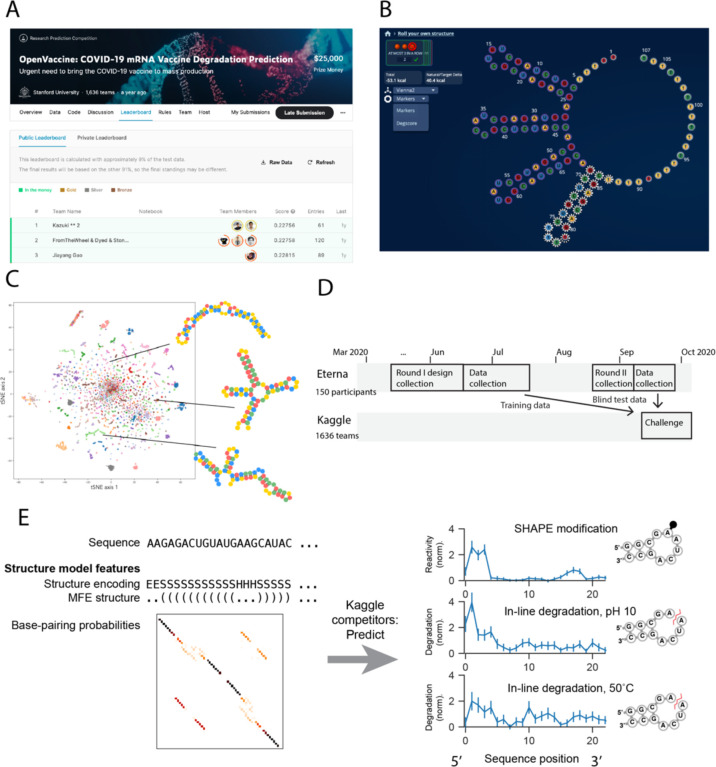
Dual-crowdsourcing setup for creating predictive models of RNA degradation. A. Screenshot of the OpenVaccine Kaggle competition, public leaderboard. B. Screenshot of an example construct designed by an Eterna participant in the “Roll Your Own Structure” challenge (“rainbow tetraloops 7” by Omei). C. tSNE^16^ projection of training sequences of “Roll-Your-Own-Structure” Round I, marker style and colors indicating 150 Eterna participants. Lines indicate example short 68 nt RNA fragments. D. Timelines of dual crowdsourced challenges. Eterna participants designed datasets that were used for training and blind test data for Kaggle machine learning competition to predict RNA chemical mapping signal and degradation. E. Kaggle participants were given RNA sequence and structure information and asked to predict RNA degradation profiles and SHAPE reactivity.

The aim of the OpenVaccine Kaggle competition (Fig. 1A) was to develop computational models for predicting RNA degradation patterns. We asked participants on the Eterna platform to submit RNA designs using a web-browser design window (Fig. 1B), which resulted in a diversity of sequences and structures (Fig. 1C). 150 participants in total (Table S1) submitted sequences. A secondary motivation was an opportunity for participants to receive feedback on RNA fragments they may wish to use in mRNA design challenges described in ref. 6. 3029 RNA designs of length 107 nt were collected in the first “Roll-Your-Own-Structure” round I (RYOS-I), which was opened March 26, 2020, and closed upon reaching 3029 constructs on June 19, 2020 (Fig. 1D).

We then obtained nucleotide-level degradation profiles for the first 68 nucleotides of these RNAs using In-line-seq^6^, a novel method for characterizing in-line RNA degradation in high-throughput for the purposes of designing stabilized RNA therapeutics. Degradation profiles were collected in four different accelerated degradation conditions, and the structures of the constructs were also characterized via selective 2’ hydroxyl acylation with primer extension (SHAPE; termed “Reactivity” below)^10, 11^, a technique to characterize RNA secondary structure. The Kaggle competition was designed to create models that would have predictive power for all five of these data types, given RNA sequence and secondary structure as input (Fig. 1E). In total, each independent construct of length 68 required predicting 5×68 values for the 5 data types. In addition to these experimental data, Kaggle participants were also provided with features related to RNA secondary structure computed from available biophysical models to use if they wished. These features included 107×107 base pairing probability matrices from EternaFold6, a recently developed package with state-of-the-art performance on RNA structural ensembles; 107-character strings representing the minimum free energy (MFE) RNA secondary structure from the more widely used Vienna RNA package; and a six-character featurization of the MFE structure developed for the bpRNA database^12^.

**Figure 2.**
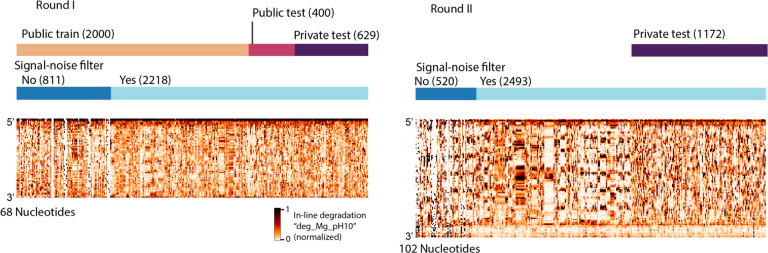
Signal-noise filtering and hierarchical clustering was used to filter the constructs designed by Eterna participants to create a test set of constructs that were maximally distant from other test constructs. Heatmaps of datatype “deg_Mg_pH10”.

We developed training and public test datasets from the RYOS-I dataset (Fig. 2). The 3029 constructs were filtered for those with mean signal-to-noise values greater than 1, resulting in 2218 constructs (Fig. 2, dark blue track). These constructs were segmented into splits of 1179 in the public training dataset, 400 constructs in the public test set, and 639 in the private test set. The sequences that did not pass the signal-to-noise filter were also provided to Kaggle participants with the according description. The RYOS-I data contained some “clusters” of sequences where Eterna players included many small variations on a single sequence (clusters visible in Fig. 1C). To mitigate the possibility of sequence motifs in these clusters biasing evaluation, we segmented the RYOS-I data into a public training, public test, and private test sets by clustering the sequences and including only sequences that were singly, doubly, or triply-clustered in the private test set. This strategy was described to Kaggle participants.

To ensure that the majority of the data used for the private test set was fully blind, we initiated a second “Roll-your-own-structure” challenge that was launched for Eterna design collection on August 18, 2020. Design collection was closed on September 7th, three days before the launch of the Kaggle challenge on September 10th. The RYOS-II wet-lab experiments were conducted concurrently with the Kaggle challenge, enabling a completely blind test for the models developed on Kaggle. The Kaggle competition was closed on October 6th. The RYOS-II was similarly clustered and filtered to ensure that the test set used for scoring consisted primarily of singly- and double-clustered constructs. Models were scored on the mean column RMSE (MCRMSE) across three data types. While the submission format required that all 5 be predicted, only three data types were collected in the testing wet-lab experiments (SHAPE; 10 mM Mg^2+^, pH 10, 1 day, 24 °C; and no Mg^2+^, pH 10, 1 day, 50 °C) and only these data types were scored: Reactivity, deg_Mg_pH10, and deg_Mg_50C. Given that useful models for degradation should be agnostic to RNA length, we designed the constructs in RYOS-II to be 34 nucleotides longer (102 vs. 68 nts) than the constructs in RYOS-I to discourage modelling methods that would overfit to constructs of length 68.

### Performance of Kaggle teams and common attributes of top-performing models.

**Figure 3.**
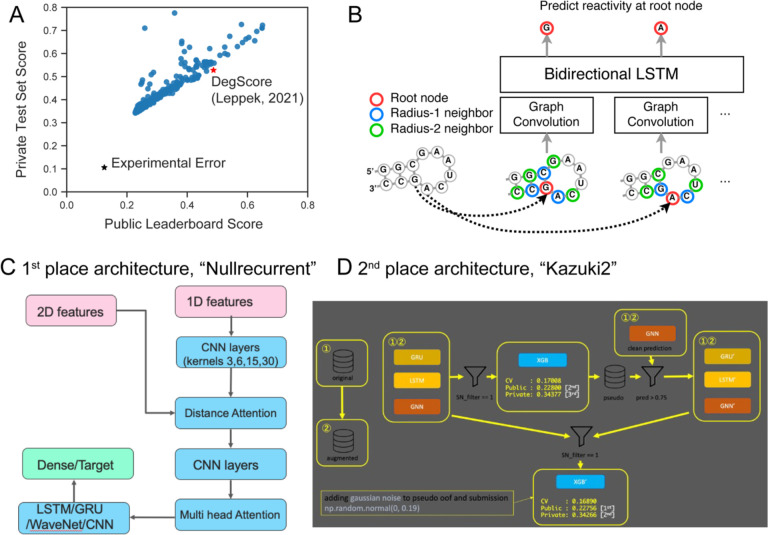
Deep learning strategies used in competition. (A) Public test vs. private test performance of all teams in Kaggle challenge. Black star: experimental error. Red star: DegScore baseline model (Leppek, 2021)^6^. (B) Distance embedding used to represent nucleotide proximity to other nucleotides in secondary structure. (C) Schematic of the single neural net (NN) architecture used by the first place solution. This solution combined two sets of features into a single NN architecture, which combined elements of classic RNNs and CNNs. (D) Schematic of the full solution pipeline for the second place solution. This solution combined single model neural networks, similar to the ones used for the first place solution, with more complex 2nd and 3rd level stacking using XGBoost^13^ as the higher level learner.

During the three week competition period, 1,636 teams submitted 35,806 solutions. Overall performance of teams vs. baseline models for RNA degradation are depicted in Figure 3A. Kaggle entries significantly outperformed the “DegScore” linear regression model for RNA degradation reported in ref. 6, by more than 50% in MCRMSE (Fig. 3A). Kaggle participants developed feature encodings beyond what was provided. One of the most widely-used community-developed featurizations was a graph-based distance embedding depicted in Fig. 3B. Many architectures used a combined autoencoder/GNN/GRU model, including the architectures of the top two solutions (Fig. 3C and Fig. 3D, respectively). Many teams cited pseudolabeling and generating additional mock data as being integral to their solutions. The machine learning practice of pseudolabeling involves using predictions from one model as “mock ground truth” labels for another model. Effective pseudolabeling usually requires a high level of accuracy of the primary model and is most frequently used with classification problems. To generate additional mock data, participants generated random RNAs as well as structure featurizations using 5 different secondary structure prediction algorithms using the package Arnie (https://github.com/DasLab/arnie) and iteratively scored based on these predictions for their model as well (see Supplement for more detailed descriptions of solutions from Kaggle teams).

### Ensembling models.

Motivated by applications to design of stabilized mRNA, we explored whether increased accuracy in modeling might be achieved by combining models. A common feature of Kaggle competitions is that winning solutions are dissimilar enough that ensembled models frequently improve predictive ability. We used a genetic algorithm to ensemble maximally 10 of the top 100 models. The score on the public dataset was used to optimize, with the final ensembled model evaluated on the private dataset. With this method, ensembling achieved a Public score of 0.2237 (compared to the best Public LB score of 0.2276) and a Private score of 0.3397 (compared to the best Private test set score of 0.3420). In comparison, averaging the outputs of the top two models gave a result of 0.2244 Public, 0.33788 Private. Blending the top two solutions with the 3rd solution did not improve the result. An estimated bound of ensembling can be found by optimizing directly to the Private ensemble score. With this method, it was possible to achieve a Private ensemble score of 0.3382 (again, vs LB 0.3420). The improvement of 0.0038 over the leaderboard for this last approach is about the distance between the 1st place and 10th place teams, and the “correct” way gives an improvement that is the distance between the 1st and 5th place teams. All these experiments suggest that most of the signal has been captured by the top two models, and that the use of further ensembling provides, at best, modest improvements. The seemingly puzzling result that the simple ensemble of the top two models outperforms the genetic algorithm blend of the top 10 (on the private test set) can be attributed to increase of the search space. The search space for the 10 different blending weights is substantially larger than for just a single weight, and it is very likely that the algorithm found a local, rather than global, minimum.

### Top models are capable of deep representation of RNA experimental motifs.

**Figure 4.**
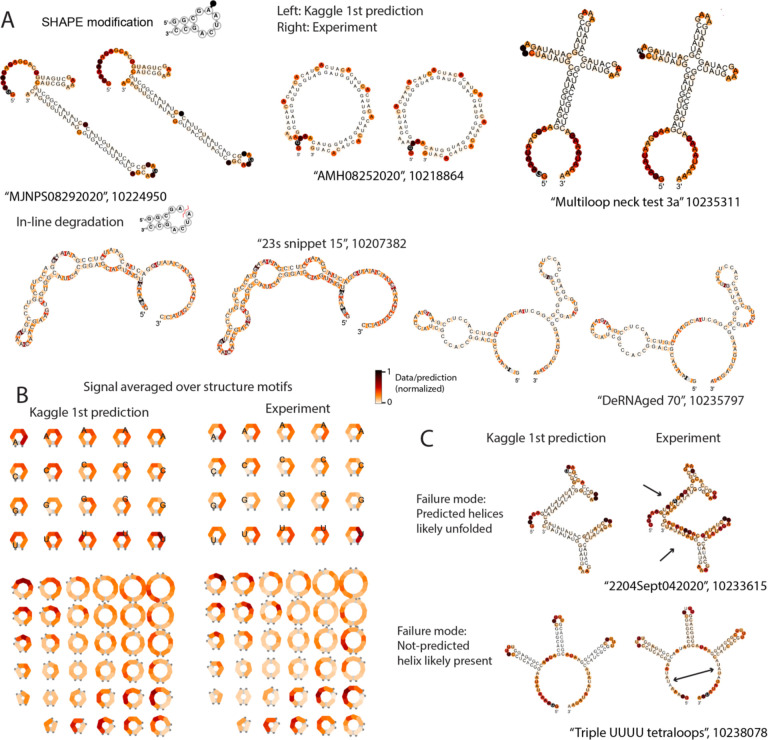
Deep-learning models can represent RNA-structure-based observables. (A) Experimental error for private dataset vs. RMSE from winning Nullrecurrent model. (B) Representative structures from the best-predicted constructs from SHAPE modification (top row) and degradation at 10 mM Mg^2+^, pH 10, 1 day, 24 °C (Deg_Mg_pH10, bottom row). (B) Nullrecurrent model predictions and experimental signal, averaged over secondary structure motifs. (C) One failure mode for prediction came from constructs whose input secondary structure features were incorrectly predicted.

We analyzed predictions from the first-place model (“Nullrecurrent”) in depth to better understand its performance. Across all nucleotides in the private test set, 41% of nucleotide-level predictions for SHAPE reactivity agreed with experimental measurements within an error that was lower than experimental uncertainty; for comparison, if experimental errors are distributed as normal distributions, a perfect predictor would agree with experimental values over 68% of data points. For Deg_Mg_pH10, 28% of predictions were within error, and for Deg_Mg_50C, 42% of predictions were. The nucleotides with the largest root mean squared error (RMSE) in the Deg_Mg_pH10 data type were any nucleotide type in bulges, and U’s in any unpaired context. Fig. 4A depicts representative constructs with the lowest RMSE for the Deg_Mg_pH10 data type out of the private test data, demonstrating that a diverse set of structures and structure motifs were capable of being predicted correctly. Aggregating the predictions from the Nullrecurrent model over secondary structure motifs (Fig. 4B) demonstrates that the Nullrecurrent predictions by motif captured patterns observed in the experimental signal in human expert analysis -- e.g., asymmetric loops exhibited higher degradation than symmetric loops. Constructs with the highest RMSE demonstrate indicators that the provided structure features were incorrect. Fig. 4C depicts two constructs with the highest RMSE for the SHAPE modification prediction. The SHAPE data for the first construct, “2204Sept042020”, has high reactivity in predicted stem areas, indicating the stems were unfolded in solution. In contrast, construct “Triple UUUU Tetraloops” has experimentally low reactivity in the exterior loop, suggesting that a stem was present. However, we found no correlation between the EternaScore, an indicator of how closely the experimental reactivity signal matches the predicted structure, and RMSE summed per construct for the private test constructs, suggesting that in general, quality of the input structure features was not a limitation in model training.

### Kaggle models show improved performance in independent mRNA degradation prediction.

**Figure 5.**
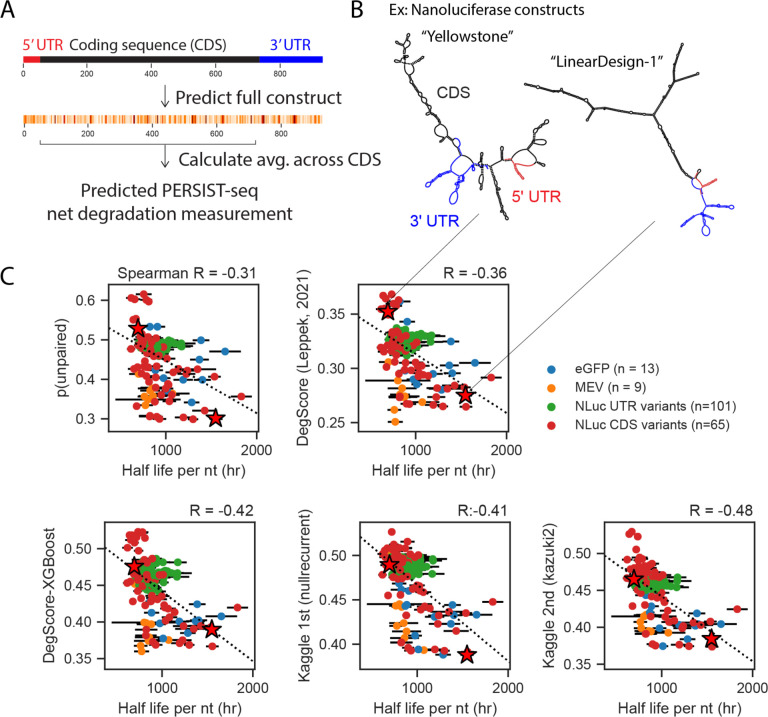
Kaggle models demonstrate improved performance in independent test of degradation of full-length mRNAs. (A) Kaggle models were tested against predictors in ref. 6 in their ability to predict net degradation measurements from the PERSIST-seq technique. The PERSIST-seq technique measures the degradation of the CDS region. Predictions were made for the full constructs, and values from the CDS region summed to compare to PERSIST-seq measurements of CDS region degradation. (B) Representative structures from ref. 6 of a destabilized mRNA (“Yellowstone”, left) and a stabilized mRNA (“LinearDesign-1”, right). (C) Four mRNA types were part of the test dataset: a short Multi-Epitope Vaccine (MEV), Nanoluciferase with varied UTRs, Nanoluciferase with varied CDS regions, and enhanced Green Fluorescent Protein (eGFP). (D) Length-normalized predictions from the Kaggle 1st place “Nullrecurrent” model and Kaggle 2nd place “Kazuki2” model show improved prediction over unpaired probabilities, the DegScore linear regression model^6^, and a version of the DegScore featurization with XGBoost^13^ training.

As an independent test for the top two Kaggle models, we ran predictions for a dataset of full-length messenger RNAs (mRNAs) from ref. 6. These data were not publicly available at the time of the Kaggle competition. The lengths of these mRNAs ranged from 504 to 1588 with a median length of 928, nearly 10-fold times longer than the longest RNA fragments used in the OpenVaccine Kaggle challenge (full dataset, attributes, and calculations in Table S2). The PERSIST-seq method was developed to determine the degradation rate of the coding sequence of an mRNA. To compare the Kaggle predictors, which make predictions per nucleotide, to this single value for degradation, we made predictions for all nucleotides in the full mRNA constructs and summed the predictions from the region that was captured in the PERSIST-seq method by reverse-transcription PCR which, in most cases, included the mRNA’s 5’ untranslated region (UTR) and coding sequence (CDS) (Fig. 5A). Carrying out predictions on the full RNA sequence and then summing over the probed window allows to account for interactions between the untranslated regions and CDS, as can be seen for two example constructs in Fig. 5B -- nucleotides in the 5’ and 3’ UTRs are predicted to pair with the CDS. We made predictions for 188 mRNAs in 4 classes: a short multi-epitope vaccine (MEV), the model protein Nanoluciferase, with one class consisting of varied UTRs and a second consisting of varied CDSs, and enhanced Green Fluorescent Protein (eGFP). We found that the Kaggle second-place “Kazuki2” model exhibited the highest correlation to fit half-lives, followed by the Kaggle 1st-place “Nullrecurrent” model (Fig. 5C), with Spearman correlation coefficients of −0.48 and −0.41, respectively. Both Kaggle models outperformed unpaired probability values from EternaFold (R=−0.31), the same kind of inputs provided to participants in the competition, the DegScore linear regression model (R=−0.36), and an additional benchmark model prepared after the Kaggle competition exploring the use of XGBoost^13^ training with the DegScore featurization (R=−0.42). An ensemble of the Nullrecurrent and Kazuki2 models did not outperform the Kazuki2 model (R=−0.45), again suggesting that the models themselves had reached their predictive potential. In comparison, resampling the measured degradation rates from within error and calculating this correlation coefficient, as a measure of the upper limit of experimental noise, resulted in a Spearman correlation of −0.95 (Table 1).

## Discussion

The OpenVaccine competition uniquely leveraged resources from two complementary crowd-sourcing platforms: Kaggle and Eterna. The participants in the Kaggle competition were tasked with predicting stability measurements of individual RNA nucleotides. The urgency of timely development of a stable COVID-19 mRNA vaccine necessitated that the competition be run on a relatively short timeframe of three weeks, as opposed to three months, which is more common with the Kaggle competitions. Kaggle competitions with relatively small datasets can be subject to serious overfitting to the public leaderboard, which often leads to a major “shake up” of the leaderboard when the results on the unseen test set are announced. In this competition the shakeup was minimal - most of the top teams were ranked close to the same position on the private leaderboard as they were on the public leaderboard. As the private leaderboard was determined on data that had not been collected at the time of the competition launch, this result suggests that the models that were developed are robust and generalizable. Furthermore, the models generalized to the task of predicting degradation for full-length mRNA molecules that were ten-fold longer than the constructs used for training. We speculate that the use of a separate, independently collected data set for the private leaderboard tests -- a true blind prediction challenge -- was important for ensuring generalizability. The winning solutions all used neural network architectures that are commonly used with modeling of 1D sequential data: recurrent NNs (LSTMs and GRUs) and 1D CNNs. The effectiveness of pseudolabeling suggests two things: more data will likely benefit any future modeling efforts, and the simple neural networks that were used have enough capacity to benefit from more data. Future directions for model development includes training such models on larger chemical mapping datasets from more diverse experimental sources^9^, and integrating into inference frameworks for RNA structure prediction^9, 14^.

## Methods

### Initial feature generation.

As a starting point for Kaggle teams, we supplied a collection of features for each RNA sequence, including the minimum free energy (MFE) structure according to the ViennaRNA 2 energy model^15^, loop type assignments generated with bpRNA^12^ (S=Stem, E=External Loop, I=Internal loop, B=Bulge, H=Hairpin, M=Multiloop, X=Dangle) and the base pair probability matrix according to the EternaFold^9^ energy model. These features were generated using Arnie (https://github.com/DasLab/arnie).

### Experimental data generation.

The first experimental dataset used in this work, for the public training and test set, resulted from the “Roll-Your-Own-Structure” Round I lab on Eterna, and had been generated previously in ref. 6.

The second experimental dataset used in this work, for the private test set, was generated for this work specifically. To produce these data, and for precise consistency with the public training and test set, In-line-seq was carried out as described in ref. 6), Methods section “High-throughput in-line and SHAPE probing on Eterna-designed RNA fragments (In-line-seq).” Chemical mapping protocols were taken from ref.^10^.

As a high-level summary of that method, DNA templates were ordered via custom oligonucleotide pool from Custom Array/Genscript, prepended by the T7 RNA polymerase promoter. Templates were amplified via PCR, transcribed to RNA via the TranscriptAid T7 High Yield Transcription Kit (Thermofisher, K0441), and the purified RNA was subjected to degradation conditions: 1) 50 mM Na-CHES buffer (pH 10.0) at room temperature without added MgCl2; 2) 50 mM Na-CHES buffer (pH 10.0) at room temperature with 10 mM MgCl2; 3) phosphate buffered saline (PBS, pH 7.2; Thermo Fisher Scientific-Gibco 20012027) at 50iC without added MgCl2; and 4) PBS (pH 7.2) at 50iC with 10 mM MgCl2. Reactions were quenched for data collection at 0 and 24 hour time points (+MgCl_2_) or 0 and 7 day time points (−MgCl_2_). In parallel, purified RNA was subjected to SHAPE structure probing conditions, and one sample was subjected to the SHAPE protocol absent addition of the 1-methyl-7-nitroisatoic anhydride reagent.

cDNA was prepared from the six RNA samples (SHAPE probed, control reaction, and four degradation conditions). We pooled 1.5 μL of each cDNA sample together, ligated with an Illumina adapter, washed, and resuspended the ligated product, which was quantified by qPCR, sequenced using an Illumina Miseq, and analyzed using MAPseeker (https://ribokit.github.io/MAPseeker) following the recommended steps for sequence assignment, peak fitting, background subtraction of the no-modification control, correction for signal attenuation, and reactivity profile normalization.

The third experimental dataset used in this work, for an independent test of the top models, was a PERSIST-seq data set for in solution stabilities of full-length mRNAs taken from ref. 6.

### Data availability.

All datasets are downloadable in raw RDAT format from rmdb.stanford.edu at the following accession numbers: SHAPE_RYOS_0620, RYOS1_NMD_0000, RYOS1_PH10_0000, RYOS1_MGPH_0000, RYOS1_50C_0000, RYOS1_MG50_0000, RYOS2_1M7_0000, RYOS2_MGPH_0000, RYOS2_MG50_0000. Kaggle-formatted train and test sets are downloadable from https://www.kaggle.com/c/stanford-covid-vaccine.

### Model availability.

Code to run the Nullrecurrent model and the DegScore-XGBoost model is available at www.github.com/eternagame/KaggleOpenVaccine. Code to use and reproduce the linear regression DegScore model is available at www.github.com/eternagame/DegScore.

## Supplementary Material

1

## Figures and Tables

**Table 1.** Results from models tested in this work on Kaggle OpenVaccine public leaderboard, private test set, and orthogonal mRNA degradation results.

	Public test set (400 constructs, 27200 nucleotides)	Private test set (1801 constructs, 162316 nucleotides)	mRNA degradation prediction from ref. 6 (188 constructs)
**Metric**	MCRMSE	MCRMSE	Spearman Correlation
Experimental error	0.12491	0.10571	−0.951
**Single Model (blind prediction)**			
DegScore	0.48724	0.52772	−0.36
Nullrecurrent	0.22758	**0.34198**	−0.41
Kazuki2	**0.22756**	0.34266	−**0.48**
**Ensembled models (post hoc)**			
Genetic algorithm (10 of top 100 selected)	**0.2237**	0.3397	
Ensemble top 2 models	0.2244	**0.33788**	−0.45
Genetic algorithm on private test set		0.3382	--

1 Bootstrapped Spearman correlation of degradation rate (resampled from experimental error) to half-life.
