# Supplementary material for: Predictive models of RNA degradation through dual crowdsourcing
Source: ArXiv. 2021 Oct 14:arXiv:2110.07531v1. Preprint. [Version 1] (PMC8528079)
Supplement: Supplementary file 1 [file NIHPP2110.07531v1-supplement-1.pdf]

Supporting information: Solution descriptions from Kaggle teams

1st Place - Jiayang Gao (also called "Nullrecurrent" model)
My final submission is a simple ensemble of 4 models, each starts with an auto-encoder pretrained GNN architecture, followed by LSTM, GRU or Wavenet layers. My model takes in two kinds of features, (1) 1D features with length n (the length of the mRNA sequence), representing features at each location of the sequence; (2) 2D features with size n^2, representing "distance" or "relationship" concepts between each pair. The strongest 1D features are the distance to the closest paired position, as well as the distance to the closest unpaired position - reactivity increases as the distance to the closest paired position becomes larger. The strongest 2D feature is the distance between each pair in the primary pairing based graph - this allows the model to capture the "neighbors" caused by pairing. One major difficulty of this problem is generalizing the model to different sequence lengths, and I use a semi-supervised approach to make my model more generalizable. In particular, I randomly generate sequences of different length, calculate their BPP matrix and pairing structure using the Arnie library, pseudo label their targets, and train them together with the original train dataset. This semi-supervised learning approach is particularly useful in this problem, for which sample size is small and labeled data is expensive to obtain.
https://www.kaggle.com/c/stanford-covid-vaccine/discussion/189620

2nd Place - Kazuki ** 2 (also called "Kazuki2" model)
The mRNA sequence is not just a linear series of data, but also constitutes a loop by pairing between specific bases. Therefore, we thought of constructing LSTM/GRU and GNN independently, and integrating the prediction results with XGBoost. The base pair probabilities (bpps) are calculated by prediction, and the calculation results differ depending on the algorithm used. Therefore, we used several algorithms in Arnie to predict base pairs, and used these algorithms as data augmentation and input multiple bpps simultaneously to improve the performance. We prepared 38 LSTM/GRU-based and 49 GNN-based bpps by changing the types of bpps and their architectures, and integrated them with XGBoost to help improve the stability of prediction. Also, since the sequences in the test set tended to be longer than those in the training data, we confirmed in preliminary experiments that the model trained by shortening the sequences in the training data (107 to 88) was applicable to sequences of the original length (107).
https://www.kaggle.com/c/stanford-covid-vaccine/discussion/189709

3rd Place - Striderl
My solution is an ensemble of various models of different structures and different training techniques. I added various LSTM/GRU/wavenet layers at the end of the AE pretrained GNN structure, in which 2 x 128 units of LSTM or GRU layers work the best for me. Besides the common features used by other teams like structure adjacency matrix and neighbor adjacency matrix, I used RNAComposer to generate 3D structures for each sample in the competition, and used predicted 3D distance to form the distance matrix. I used Arnie to predict other possible base pairs as data augmentation. Besides, another augmentation I used was to reverse the sequence and targets. The two augmentations quadruple the size of the original data. I also used pseudo labeling technique to iteratively improve my best single model.

4th Place - FromTheWheel & Dyed & StoneShop
The 4th place solution is a blend of 4 different models, in which the RNN layers were varied (LSTM+LSTM, LSTM+GRU, GRU+LSTM and GRU+GRU). We represent the RNA sequences as graphs

where each base corresponds to a node. The network then learns a representation for each of these graphs and passes this representation through bi-directional RNN layers to obtain a sequence of predicted targets. Both the edge and node features were derived from the given sequence and provided Base Pairing Probability (BPP) matrix. One-hot-encoded bases (A, G, C, or U), one-hot-encoded positional feature (the remainder of the base index divided by 3), one-hot-encoded loop types, loop type probabilities (CapR) and BPP sum and number of zero's were beneficial node features. The distances (manhattan) between bases, normalized by sequence length and whether there is a base-pairing indicated in the structure were used as edge features. All these features were also inferred for newly generated BPP matrices, generated with 6 libraries available within the ARNIE software package. The new information derived from the CONTRAfold library was the most useful for this task, followed by RNAsoft, RNAstructure and Vienna. We also tried to use 3D angle information (binned and then categorically encoded) extracted with AMIGOS: this boosted our simple LSTM architecture used to fast check the feature importance but deteriorated the performance of our final model.

5th Place - tito
My solution is a simple ensemble of GNN-based model and GRU/LSTM-based model. The features are not significantly different from those used by the other teams. I focused mainly on augmentation. (1) I used eternafold, vienna, nupack, contrafold and rnasoft to extract structure and loop_type. These backend engines are used to extract additional bpps too. Especially eternafold and contrafold worked well. (2) In this competition, the sequence length of the test data was longer than that of the training data, so we added a dummy sequence to the training data. (3) I added reversed sequence to training data. I think augmentation was important in this competition.

6th Place - nyanp
The data in this competition is very unique in two ways: 1) the sequence length is different between the training data and the private test data, and 2) the sequence has long-term dependencies via pairing. My NN model is constructed by stacking 1D SE-ResNet Layer and Graph Convolution Layer in order to make the model invariant to sequence length and to capture the long-term dependency. The Graph Convolution Layer is computed by a simple sum of products of the BPP matrix or adjacency matrix and the sequence feature vector. The best single model was ranked 42nd (0.35045) on the private leaderboard, while it was 513th (0.24371) on the public leaderboard. This indicates that my model is more robust to changes in sequence length compared to the other participants. Best ensemble achieved MSRMSE's of 0.23069/0.34538 on the public/private leaderboard by combining 4 different architectures.
https://www.kaggle.com/c/stanford-covid-vaccine/discussion/189241
https://www.kaggle.com/nyanpn/6th-place-cnn-gcn

7th Place - One architecture
Main features for my solution used were base pairing probability matrix, nucleotide sequence, structure, and loop type. Additionally, an inverse distance matrix (nucleotides at position i and j have distance |i-j| between them) was added to the base pairing probability matrix before inputting it as a bias for the self-attention matrix. The conventional type of positional encoding as detailed in the original transformer or learnable positional encoding were not used; instead, position was encoded by the inverse distance matrix. Further, 5 secondary structure packages were used to generate pairing probability matrix, structure, and loop type at 37 and 50 C, resulting in 10 sets of features for each sequence. The architecture used was almost identical to bert aside from the 1D/2D convolution/deconvolution layers (without padding). The core module (ConvTransformerEncoder) was constructed as follows: (1) 1D convolution on the sequence of encodings and 2D convolution on the bpp feature map. (2) self-

attention with bpp feature map as additive bias (3) position wise feedforward network. (4) 1D deconvolution on the sequence of encodings and 2D deconvolution on the bpp feature map. All available sequences were used to pretrain (unsupervised) models on randomly mutated or masked (with NULL token) sequence retrieval loss (basically just softmax to retrieve correct nucleotide/structure/loop). For convenience, two linear decoders were initialized before pretraining, one for sequence retrieval, and another for degradation predictions later on. The Ranger optimizer was used with a flat and anneal schedule. Some sequences were excluded during training on degradation targets based on signal to noise threshold (0.25, 0.5, or 1). My biggest discovery from this competition is that the vanilla positional encoding used in the original transformer paper does not generalize well to this task at least. It seems that the type of positional encoding used in most transformers does not adequately describe the concept of position, which is fine for NLP because I believe order and position of words are not as important as for RNA. The vanilla positional encoding is more of an absolute positional encoding, whereas the inverse distance basically encoders relative position in a very simple way that generalized better to longer sequences. Best ensemble achieved MSRMSE's of 0.23056/0.34550 on the public/private leaderboard.

8th Place - ishikei
My solution is an ensemble of GRU/LSTM and GNN. Each model is AE pretrained with all data. For features, bpps was augmented with different temperature parameters (T=37, 50) using the ARNIE package (vienna, nupack, rnastructure, rnasoft, eternafold, contrafold). I also added the shannon entropy at each base position. Because all of the data in this competition are predicted values except for the sequence, I think it would have been effective to use an ensemble of bpps predictions from various algorithms. At high temperature: Since the secondary structure of RNA is temperature-dependent, I think it was effective to use bpps with T changed as input. At high pH: Alkaline hydrolysis of RNA can occur at any position in the sequence (probably) as well, so I think the prediction itself is difficult. https://www.kaggle.com/c/stanford-covid-vaccine/discussion/190314

9th Place - Keep going to be GM
The big issue of this contest is that the train and test RNA sequence lengths are different, and the data contains noise. The RNA sequence length used for training was 107, and the final ranking was obtained for 130 sequences. Different models with fluent feature engineering to enhance the generalization of predictions. Because RNA can have both graph and sequence, traditional recurrent neural networks (LSTM, GRU), transformer and graph-neural networks were applied. For feature engineering, (A, G, C, or U) were represented by embedding layers. And, various (N, N) adjacency matrices called bpps, which are probabilities of being linked between nucleotides are calculated with various softwares, such as CONTRAFold, RNAFold. The statistical features such as ratio of (A, G, C, or U) in sequence are added. has are also included.I created the (N, N) matrix for fixed attention in various ways. Bpps was created using several packages. The distance matrix was augmented using gaussian. With these features, dozens of models were created by differing hyperparameters of sequential blocks and graph convolution layers. https://www.kaggle.com/c/stanford-covid-vaccine/discussion/189845

11th Place - Social Distancing Please
Our solution is an ensemble of multiple models. There are mainly 2 types of models. The first type is a combination of 1D Convolution Layers, Graph Convolution Layers, and RNN Layers. The second type is a combination of WaveNet layers and RNN Layers. The most powerful features are the adjacency matrices constructed by the given structure sequence and the given base-pair probabilities. The adjacency matrices are used for the Graph Convolution Layers. Another useful trick we have used is to apply a lower training weight to the top 6 sequence position, it is because the sequence start is similar across

the different sequences. We also used 2 different Linear Regression models to ensemble the predictions in different sequence positions that are seqpos[:6] and seqpos[6:].

13th Place - The Machine
The main idea behind our approach is generating bpp matrices from all the libraries included in Arnie, training a model from the output structures of each library and finally creating an ensemble of all the trained models. Although each library provides sub optimal bpp, their consensus provides a better solution. We also included several architectures in the ensemble and the best of them consisted of 1D convolution, static graph convolution and bi-directional LSTM layers. A static graph convolution layer processes each two connected nucleotides in the predicted secondary structure (zeros added when a nucleotide isn't connected). All our models were trained on all the data using self supervised learning then fine-tuned on the training data only using supervised learning.
